# Estimating Neural Control from Concentric vs. Eccentric Surface Electromyographic Representations during Fatiguing, Cyclic Submaximal Back Extension Exercises

**DOI:** 10.3389/fphys.2017.00299

**Published:** 2017-05-16

**Authors:** Gerold R. Ebenbichler, Lena Unterlerchner, Richard Habenicht, Paolo Bonato, Josef Kollmitzer, Patrick Mair, Sara Riegler, Thomas Kienbacher

**Affiliations:** ^1^Department of Physical Medicine, Rehabilitation and Occupational Medicine, Medical University of ViennaVienna, Austria; ^2^Karl-Landsteiner-Institute of Outpatient Rehabilitation ResearchVienna, Austria; ^3^University of Applied Sciences, Business InformaticsVienna, Austria; ^4^Department of Physical Medicine and Rehabilitation, Harvard Medical School, Spaulding Rehabilitation HospitalBoston, MA, USA; ^5^Technical School of EngineeringVienna, Austria; ^6^Department of Psychology, Harvard UniversityCambridge, MA, USA

**Keywords:** electromyography, muscle fatigue, concentric exercise, eccentric exercise, time-frequency analysis

## Abstract

**Purpose:** To investigate the differences in neural control of back muscles activated during the eccentric vs. the concentric portions of a cyclic, submaximal, fatiguing trunk extension exercise via the analysis of amplitude and time-frequency parameters derived from surface electromyographic (SEMG) data.

**Methods:** Using back dynamometers, 87 healthy volunteers performed three maximum voluntary isometric trunk extensions (MVC's), an isometric trunk extension at 80% MVC, and 25 cyclic, dynamic trunk extensions at 50% MVC. Dynamic testing was performed with the trunk angular displacement ranging from 0° to 40° and the trunk angular velocity set at 20°/s. SEMG data was recorded bilaterally from the iliocostalis lumborum at L1, the longissimus dorsi at L2, and the multifidus muscles at L5. The initial value and slope of the root mean square (RMS-SEMG) and the instantaneous median frequency (IMDF-SEMG) estimates derived from the SEMG recorded during each exercise cycle were used to investigate the differences in MU control marking the eccentric vs. the concentric portions of the exercise.

**Results:** During the concentric portions of the exercise, the initial RMS-SEMG values were almost twice those observed during the eccentric portions of the exercise. The RMS-SEMG values generally increased during the concentric portions of the exercise while they mostly remained unchanged during the eccentric portions of the exercise with significant differences between contraction types. Neither the initial IMDF-SEMG values nor the time-course of the IMDF-SEMG values significantly differed between the eccentric and the concentric portions of the exercise.

**Conclusions:** The comparison of the investigated SEMG parameters revealed distinct neural control strategies during the eccentric vs. the concentric portions of the cyclic exercise. We explain these differences by relying upon the principles of orderly recruitment and common drive governing motor unit behavior.

## Introduction

Progressive trunk muscle resistance training is widely recommended to increase back muscle strength, endurance and power, which in turn allows one to maintain pain-free functional performance of trunk movements and to build injury resilience (Scharrer et al., 2012; Watson et al., 2015; Steffens et al., 2016). Evidence-based training recommendations encourage the performance of a set number of repetitions of dynamic, cyclic contractions for both healthy individuals and patients (Garber et al., 2011). However, it is unclear how the central nervous system (CNS) controls the motor units that are recruited during the concentric vs. the eccentric phases of cyclic exercises of extension of the trunk, hence making it difficult to predict the effects of exercise-based interventions.

The production of force by skeletal muscles is dependent on the interactions among contractile proteins, the number and firing rate of active motor units (MUs), the geometrical distribution of active muscle fibers, the muscle length and the change in muscle length during muscle contractions (Herzog et al., 2015; Duchateau and Enoka, 2016). Concentric contractions require muscles to generate a force that exceeds the load to be displaced. In contrast, during eccentric contractions, muscle fibers have a greater intrinsic force capacity (Herzog et al., 2015) and the generated force is smaller than the load that is to be resisted. Prior work has suggested that eccentric contractions are marked by the recruitment of fewer MUs that fire at lower rates than during concentric contractions, if the muscle length vs. force relationship is comparable (Pasquet et al., 2006; Kallio et al., 2013; Duclay et al., 2014). Furthermore, recruitment and de-recruitment of MUs during non-isometric, cyclic contractions have been shown to follow a hierarchical order according to the MU size and the principle of common drive of MUs (De Luca et al., 2015), which is the synchronous modulation of the firing rate of all active MUs of a given muscle (De Luca and Erim, 1994). Such modulation corresponds to a simultaneous increase/decrease in the excitation of all MU pools. This observation suggests that some similarities in MU behavior are likely to mark the eccentric and concentric muscle contractions during slow cyclic exercises.

When compared to limb muscles, the back extensor muscles are composed of many different muscle fascicles that are innervated separately (Mannion, 1999). These muscle fascicles are topographically organized according to the anatomical location, i.e., the number of vertebral segments they span. Besides, they differ in geometrical orientation and direction of torque production as well as in their global function as primarily providing either joint stability or movement control (Ebenbichler et al., 2001). Moreover, the activation of these fascicles may vary according to the posture and the curvature of the spine (Dolan and Adams, 1998; Park et al., 2014). The erector spinae muscles are known to be composed of a relatively high percentage of slow twitch muscle fibers that are larger in cross section than the fast twitch fibers of either the medial or lateral back extensor muscles, and they likely produce higher twitch contraction forces (Mannion, 1999). Differences in muscle spindle density between the deep, mono-segmental and the more superficial and/or lateral oligo- to poly-segmental back extensor muscles (Amonoo-Kuofi, 1982, 1983) may influence the thresholds of recruitment and de-recruitment and the maximum firing rates of the MUs from different parts of the back extensors in a significant way as indirectly evidenced from extremity muscles (De Luca and Kline, 2012; Duclay et al., 2014; Kline and De Luca, 2016) and would modulate the neural control of concentric vs. eccentric contractions in a differential way. Based on all these special structural and functional anatomic features of the back extensors, it remains unknown whether the neuromuscular control strategies that were found to be associated with concentric and eccentric contractions in upper and lower limb muscles would also hold true for the back extensors.

The analysis of surface electromyographic (SEMG) data could shed light on the neuromuscular control mechanisms underlying concentric and eccentric extensions of the trunk. SEMG data recorded during voluntary muscle contractions is regarded as the spatio-temporal superposition of the motor unit action potential (MUAP) trains of active MUs. The physiologic correlates contributing to the SEMG data amplitude are mainly the number of active MUs and their firing rates, whereas changes in the frequency content of the SEMG data correlate with changes in the conduction velocity of the active muscle fibers (De Luca, 1997).

During sustained or repetitive concentric contractions at high target force, changes in the amplitude of the SEMG data reflect an increase in firing rate and progressive MU recruitment required to compensate for the fatigue-related output failure of fast-fatiguing MUs (Bigland-Ritchie and Woods, 1976; De Luca, 1997); Correspondingly, the frequency content of the SEMG data shows a compression toward the lower frequencies that well correlates with a decrease in muscle fiber conduction velocity (Brody et al., 1991; Kupa et al., 1995). These observations suggest that changes in the characteristics of SEMG data can be used to estimate molecular correlates of muscle fatigue. For instance, changes in the frequency content of SEMG data collected during sustained extensions of the trunk have been shown to change at a rate that is proportional to the level of contraction in the back extensor muscles with levels lower than 40% of maximum inducing almost no fatigue (Roy et al., 1989; Oddsson and De Luca, 2003). Moreover, *in vitro* studies have demonstrated a close relationship between muscle fiber size, type and the median frequency of the SEMG suggesting that relative fatigue-related changes in the SEMG spectrum could indicate neural control properties of muscle fibers of the activated muscle (Kupa et al., 1995).

Traditional techniques (e.g., Fourier analysis) to derive the frequency content of SEMG data are only suitable for the analysis of stationary time series. SEMG data collected during dynamic contractions is non-stationary. Hence, techniques suitable for the analysis of non-stationary time series need to be applied to analyze SEMG data collected during dynamic contractions (Knaflitz and Bonato, 1999). Time-frequency representations of the Cohen class and wavelet-based transformations have been shown to allow one to reliably monitor muscle fatigue during dynamic contractions (Ebenbichler et al., 2002; Larivière et al., 2010, 2011).

To the best of our knowledge, no previous research has been focused on analyzing SEMG data collected during cyclic, submaximal, repetitive extensions of the trunk comparing the concentric and the eccentric portions of the exercise. Hitherto, only one study has used the above-mentioned transformations for the analysis of the time-frequency content of SEMG data collected during purely concentrically-generated vs. purely eccentrically-generated knee extensions at maximal effort (Molinari et al., 2006). The authors achieved this goal by using a transformation of the Cohen class. They showed a compression toward the lower frequencies for both the concentric and the eccentric portions of the task. However, the authors reported a more pronounced compression toward the lower frequencies during the concentric contractions than during the eccentric contractions.

The study herein presented investigated the amplitude and frequency characteristics of the SEMG data collected during the concentric and eccentric portions of an exercise consisting of cyclic, submaximal extensions of the trunk at moderately high effort, sufficient to induce “electromyographic” fatigue (Oddsson et al., 1997). Movement velocity, range of motion, and the external load were consistent during the eccentric and concentric portions of the exercise.

We anticipated observing lower amplitude of the SEMG data recorded during the eccentric vs. the concentric portions of the first cycles of the trunk extension exercise because eccentric contractions require a lower effort in motor neuronal activity to resist an external load than concentric contractions to move the same load. Consequently, we hypothesized that the experimental data would have shown more pronounced fatigue related changes in SEMG amplitude during the performance of the concentric vs. the eccentric portions of the exercise would be expected to occur. Furthermore, we hypothesized that we would have observed similar frequency characteristics of the SEMG data during the eccentric vs. the concentric portions of the first cycles of the trunk extension exercise. We formulated this hypothesis based on the assumption that differences in MU pools recruited during the concentric vs. the eccentric portions of the exercise would have had a negligible effect on the frequency content of the SEMG data. Finally, we considered the potential changes in frequency content of the SEMG data during the exercise. We observed that, if a significantly higher number of high-threshold MUs happened to be recruited during the concentric vs. the eccentric portions of the exercise, then the rate of change in frequency content of the SEMG data would be higher during the concentric vs. the eccentric portions of the exercise. Vice versa, if a similar number of high-threshold MUs happened to be active during the eccentric vs. the concentric portions of the exercise, then the rate of change in frequency content of the SEMG data would be similar during the eccentric vs. the concentric portions of the exercise. As muscle fiber structures of the back extensors are known to differ between males and females (Mannion, 1999), and the composition of the back extensor muscles as well as the size of the motor units likely depends on age (Kienbacher et al., 2014a; Hunter et al., 2016), we further examined whether sex and age would affect the characteristics of the SEMG data collected during concentric vs. eccentric portions of the task.

## Methods

### Participants

A total of 87 asymptomatic volunteers (46 men, 41 females, between 18 and 90 years of age) were enrolled in the study. Volunteers were recruited via personal contacts, presentations at community centers for older adults, presentations at companies in the area close to the Karl-Landsteiner institute of outpatient rehabilitation research, and personal contacts with staff at an outpatient rehabilitation institute.

Physical Medicine and Rehabilitation physicians screened all participants. Volunteers were considered eligible to participate in the study if they were healthy, performed normal physical activity (but did not participate in competitive sports more than 2 times per week) and were not affected by any conditions that could preclude their participation in vigorous exercise. Volunteers were not enrolled in the study if (1) they were unable to follow verbal instructions, (2) experienced more than 5 mild back pain episodes or episodes leading to referral for physical treatment (VAS > 30) lasting more than 2 days within the past year, (3) had a history of spine surgery, (4) were pregnant, (5) were affected by any medical condition that could interfere with maximum strength or sub-maximum endurance testing, or (6) had a BMI exceeding 35 kg/m^2^.

The study protocol was approved by the Ethics' Committee of the city of Vienna. Before inclusion, all participants provided written informed consent. The data collection was carried out in accordance with the Declaration of Helsinki. Participants received a financial compensation for the time spent participating in the study.

### Experimental protocol

#### Overview

Each study participant completed the following procedures. (1) Collection of basic anthropometric measures and administration of questionnaires to assess subjects' motivation and physical activity level. (2) Performance of warm-up and maximum isometric back extension tests using a dynamometer (F110 extension; DAVID® health solutions, Helsinki, Finland). (3) Rest for approximately 20 min, during which the SEMG electrodes were positioned. (4) Performance of one sustained isometric back extension for 30 s at 80% of the maximum voluntary contraction (MVC) level performed with the Technogym dynamometer “Total Trunk” (TechnoGym®, Italy). (5) Rest for approximately 5 min and performance of a task consisting of cyclic extensions of the trunk at 50% MVC on the Technogym dynamometer “Total Trunk” (TechnoGym®, Italy). All tests were supervised by 3 experienced examiners (CS, MW, RH) and a certified clinical psychologist (BP). If the SEMG data recorded during the experiment was affected by significant artifacts, a second experimental session was conducted 1 or 2 days after the first one and all experimental procedures were repeated a second time.

### Instrumentation (equipment and tests)

#### Back dynamometer

The maximum isometric back extension torque was required to set submaximum loads for the electromyographic fatigue tests. Each study volunteer was measured using a dynamometer (F110 extension; DAVID® health solutions, Helsinki, Finland). The device has been described in a previous publication (Kienbacher et al., 2014b). It consists of a “hip locking mechanism” comprising 5 components: footplates adjustable according to the length of the lower leg, knee pads adjustable according to the thigh length, a pelvic belt, an adjustable seat to accommodate for subjects of different height, and a dorsal pad to be positioned on the back of the subject. In order to perform the maximum back extension test, study participants were instructed to sit with the axis of rotation of the knees parallel to the seat, the trunk flexed forward at about 30°, and the arms relaxed and positioned to the side of the trunk.

#### Back extension device used to study muscle fatigue in static and dynamic conditions

In order to obtain reliable SEMG recordings from the back extensor muscles, the electromyographic muscle fatigue studies during the performance of static and dynamic contractions were carried out using the “Total Trunk” (TechnoGym®, Italy) device. This device is more suitable to collect reliable SEMG recordings than the device that we used to collect the maximum isometric back extension torque data. It is equipped with a dorsal sacral pad instead of a back pad thus providing better access to the back extensor muscles than the DAVID® device. Otherwise the Total Trunk machine is built in a way that is similar to the DAVID® device. It is equipped with footplates adjustable according to the length of the lower leg, knee pads adjustable according to the thigh length, a pelvic belt, and an adjustable seat to accommodate for subjects of different height. The static study consisted of a sustained isometric extension of the trunk at 80% MVC. The dynamic study consisted of cyclic trunk extensions at 50% MVC. A 50% MVC was chosen because a relatively large proportion of the motor units would be recruited in the back extensors, blood supply to the back muscle fibers likely be partially suppressed only and at this load was demonstrated to be the minimum threshold to induce spectral SEMG muscle fatigue in the back extensors (Oddsson et al., 1997; Oddsson and De Luca, 2003).

#### SEMG system

SEMG signals were recorded using active double-differential electrodes that integrated triaxial accelerometers (Trigno, DelSys®, Boston, MA, USA). Before positioning the electrodes, the skin was cleaned with alcohol wipes and, if needed, the area of interest was shaved. Electrodes were positioned bilaterally on the multifidus muscle at L5, the longissimus dorsi muscle at L2, and the iliocostalis lumborum muscle at L1. The electrodes were positioned according to the SENIAM project recommendations (Hermens et al., 1999) and consistently with previous studies with focus on back muscles (Lariviere et al., 2002). All the sensors were attached to the skin using a double-sided adhesive interface. Members of the research team with significant experience in performing EMG recordings were responsible for correctly positioning the electrodes. Anatomical landmarks were used to position the SEMG electrodes correctly. The SEMG signals were acquired with a total effective gain of 909 V/V ± 5%, a bandwidth of 20-450 Hz and a baseline noise <0.75 μV (root mean square—RMS). The SEMG signals were sampled at 2,000 Hz using a 16-bit A/D converter and the EMG Works® acquisition software (DelSys®, Inc., Boston, MA, USA).

#### Accelerometric signal

A triaxial accelerometer (Trigno, DelSys Inc®, Boston, MA, USA) was attached to the lever arm of the dynamometer in a standardized position and used to monitor the range of movement and movement velocity during the exercise of cyclic extensions of the trunk. The triaxial accelerometer acquired preamplified signals with a dynamic range of ±1.5 g and a bandwidth from DC to 50 Hz. The accelerometer signals were sampled at 160 Hz with a resolution of 8 bits using the EMG Works® acquisition software.

#### Questionnaires

Study participants completed the International Physical Activity Questionnaire (IPAQ) (Craig et al., 2003). Besides, at the end of the 50% MVC repetitive trunk extension exercise, subjects rated their perceived back muscle exertion level using a Borg scale ranging from 0 (no perceived exertion at all) to 10 (most severe perceived exertion ever experienced).

### Test procedures

#### Maximum back extension test

After positioning the subjects for testing with the F110 extension device (DAVID® health solutions, Helsinki, Finland), members of the research team instructed them to perform a warm-up exercise consisting of extensions of the trunk at very low force level. These trunk extension exercises allowed subjects to familiarize themselves with the equipment and the experimental procedures. Thereafter, subjects performed 2 consecutive isometric maximum voluntary contractions (MVCs) under the supervision of research personnel. If the 2 tests varied by more than 10%, or if the peak moment was achieved later than 3 s after the onset of the contraction, additional trials were performed until a consistent (across trials) maximum force output was achieved. The best value observed during these tests was recorded and stored. Verbal instructions and encouragement were standardized.

#### 80% MVC trunk extension test

After placing the SEMG electrodes, study participants were instructed to position themselves for testing with the “Total Trunk” device (TechnoGym®, Italy) using the same settings that were used for the DAVID® device. Subjects maintained a 30° anteflexed trunk position for at least 30 s while performing a contraction at 80% MVC level. The MVC force output was derived from the best maximum trunk extension moment obtained with the DAVID® device. The torque generated by the subject was calculated as the mathematical product of the force recorded by the load cell of the dynamometer and the distance of the back restraint from the load cell.

#### Cyclic exercise of trunk extensions

**Figure 1:**
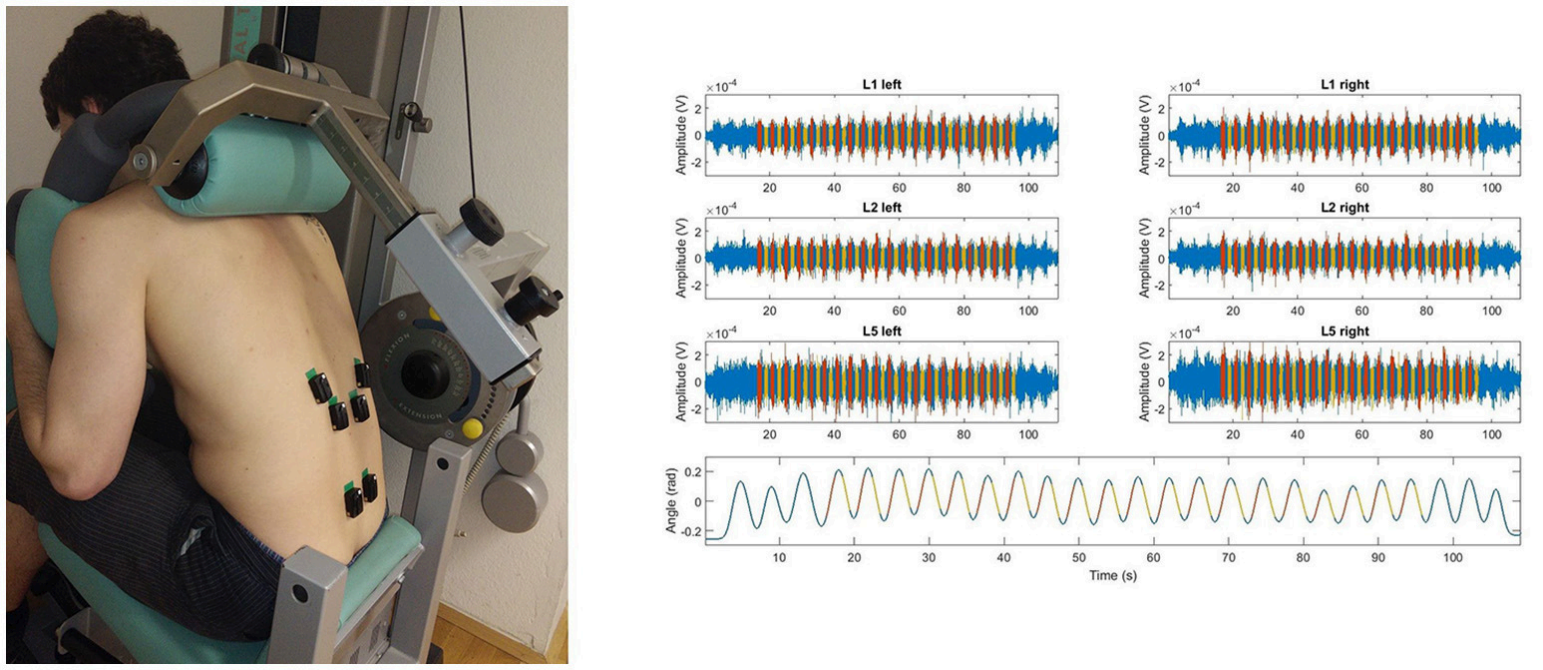
**The picture (left) illustrates the positioning of the SEMG sensors (Trigno, DelSys Inc.®, Boston, MA, USA) attached when the testees performed the cyclic 50% submaximum exercise using the “Total Trunk” back exercise device (TechnoGym®, Italy)**. The figures on the right side show the raw SEMG recorded from 6 electrodes and the trunk angular displacement estimated from the accelerometric signal recorded during the cyclic exercise; SEMG and accelerometric signal corresponding to the concentric portion of the cyclic exercise (red), that to the eccentric portion (yellow), SEMG signal not used for further data processing (blue). Data is shown for a representative subject.

The cyclic exercise of trunk extensions was performed from a seated position with arms and hands positioned in a standardized way (Figure 1) using the “Total Trunk” device (TechnoGym®, Italy). After a few practice trials without external load, volunteers performed cyclic extensions of the trunk from the upright position of 0° and the forward flexed position of 40°. The eccentric portions of the exercise consisted of movements from 0° to 40° of trunk flexion during which study volunteers resisted the external flexion torque generated by the dynamometer. The concentric portions of the exercise consisted of movements from 40° to 0° during which study participants overcame the resistive torque of the dynamometer. Subjects were asked to perform a total of 25 cycles of extension of the trunks with a load equal to approximately 50% MVC. Subjects were encouraged to move at the pace set by a metronome. Consistently, each cycle of the exercise lasted approximately 4 s. If subjects could not perform 25 repetitions or did not perform consistently all the repetitions of the exercise, the test was stopped. An exercise was considered complete if the subject performed at least 15 cycles with consistent biomechanics. An illustration of the experimental set up is provided in Figure 1.

#### Subjective rating of muscle fatigue

Immediately after the cyclic exercise of trunk extensions, study participants were asked to rate the perceived exertion of the back muscles using the Borg VAS scale.

### Data analysis

MATLAB routines (The MathWorks, Inc., Natick, MA, USA) were used to process the SEMG data collected during the experiments. The SEMG data was filtered using a 20 Hz high-pass Butterworth filter and a 500 Hz low-pass Butterworth filter. RMS-SEMG estimates were derived from the 2nd to the 5ths of the sustained 80% MVC contractions, as described in a previous manuscript (Kienbacher et al., 2014b). The RMS-SEMG values derived from the 80% MVC contractions were used to normalize the RMS-SEMG values derived from the analysis of the data collected during the cyclic exercises.

**Figure 2:**
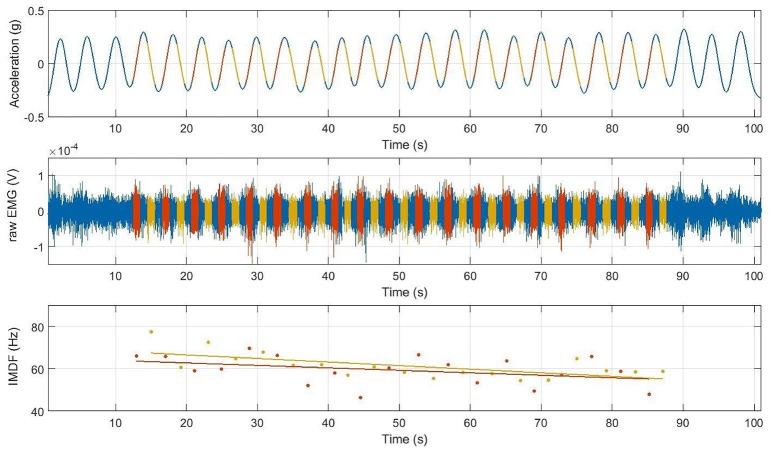
**Trunk angular displacement (top)**, raw SEMG data **(middle)**, and IMDF-SEMG estimates **(bottom)** over the duration of the full cyclic trunk extension exercise; SEMG processed form the concentric portion (red), from the eccentric portion (yellow); EMG signal not processed (blue). Data is shown for a representative subject. Note that at the beginning and at the end of the exercise the first 2–3 and last 2–3 cycles were omitted.

SEMG signals recorded during the cyclic exercises were segmented to select the concentric and the eccentric portions of the exercise. Accelerometer data recorded using the sensor positioned on the lever arm of the Total Trunk device was used to facilitate the data segmentation. We excluded portions of the exercise during which the acceleration of the trunk was disproportionally large. On average, the segmentation led to 1 s intervals for each portion of the cycle. Thus, movement velocity and muscle length-torque characteristics were similar during the SEMG segments considered to analyze the concentric and the eccentric portions of the exercise (see Figure 2).

The RMS-SEMG and the instantaneous median frequency (IMDF-SEMG) were estimated for each data segment and each electrode recording site. IMDF-SEMG estimates were derived using a Cohen class time-frequency transformation (Cohen, 1995). Software routines to derive the IMDF-SEMG estimates were implemented as in previous studies (Bonato et al., 2001). Localized muscle fatigue was quantified by estimating the least-squares regression line fitting the IMDF-SEMG estimates derived for each cycle of the exercise of trunk extensions (see Figure 2). The slope of the regression line was considered as a measure of the progression of localized muscle fatigue.

The linear regression of the RMS-SEMG estimates derived from the SEMG data collected during the cyclic exercises was used to estimate the rate of change in RMS-SEMG values, which in turn was considered a measure of localized muscle fatigue. Consistently with the work by Larivière et al. (2010), the RMS-SEMG and the IMDF-SEMG data was normalized by the initial values of the parameters (i.e., intercept with the y-axis). In order to improve upon the reliability of the proposed measures, the results from the analysis of the data collected with the three electrodes at the levels L1, L2, and L5 were averaged (Lariviere et al., 2002).

#### Accelerometer signal analysis

**Figure 3:**
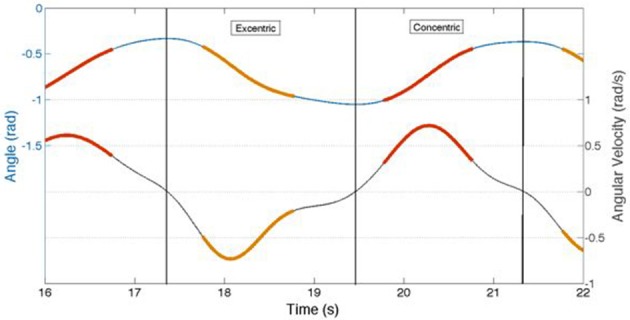
**This figure illustrates the concentric and eccentric angular velocities and respective acceleration velocities obtained from those phases where the EMG signal had been extracted during the cyclic back extension exercise at 50%MVC paced at 4 s/cycle**. Red and orange line segments represent the angular velocity (upper line) and acceleration (lower line) for either the concentric (red) or eccentric (orange) phase of the cycle from which the EMG signal had been extracted. Note that the angular acceleration velocities were non-linear in either the concentric or eccentric contraction phase, indicating the necessity of analyzing the frequency content of the EMG signal using time frequency analyses.

The orientation of the lever arm of the Total Trunk device was considered as a proxy of the trunk angular displacement. Hence, the trunk angular displacement was derived from the acceleration measured along the vertical axis (i.e., z-axis) of the accelerometer positioned on the Total Trunk device. The orientation α was estimated using the formula α = asin(acc/g), where *asin* denotes the arcsine (inverse sine function), *acc* is the acceleration measured along the z-axis of the accelerometer positioned on the Total Trunk device, and *g* is the gravitational acceleration. This is an approximation of the actual orientation of the trunk angular displacement that we deemed suitable for the purpose of segmenting the SEMG data and comparing different cycles of the exercise. An approximation of the trunk angular velocity was derived from the estimated trunk angular displacement by low-pass filtering the angular displacement time series with an 8th order low-pass Butterworth filter with a cut-off frequency of 1 Hz and by estimating its first derivative. Figure 3 shows an example of the estimated trunk angular displacement and velocity time series for a randomly selected subject and portion of exercise.

### Statistical analysis

The following dependent variables were used in the analysis: MVC as a measure of trunk extensor strength; mean normalized (80% isometric back extension) RMS-EMG and absolute IMDF-SEMG values derived from the initial values of linear regression models; mean RMS-SEMG and IMDF-SEMG slopes normalized by the RMS-SEMG and IMDF-SEMG initial values.

Secondary analyses were carried out on the phase duration and the acceleration of the concentric and eccentric portions of the cyclic exercise, and the fatigue scores reported by study participants immediately after completion of the cyclic exercise of trunk extensions.

All statistical analyses were carried out using the R [2016] environment for statistical computing. Inspection of the analyzed variables showed clear deviations from normality, especially in terms of skewness. Thus, we used tests based on comparisons of median values (Wilcox, 2012) which are also robust to violation of the assumption of homoscedasticity. These tests were implemented by relying on the WRS2 package of the R environment for statistical computing (Mair and Wilcox, 2017). The one-way version of the median test was used to test for differences in the concentric vs. the eccentric portions of the exercise. The two-way version of the median test was used to test for the effects of age and gender. The level of significance was set at *p* < 0.05.

## Results

Table 1 summarizes the characteristics of the study participants, namely age, height, weight, BMI, back extension strength measured using the above-mentioned trunk dynamometer, perceived exertion as rated by the subjects at the end of the cyclic exercise test using the Borg scale, and the total physical activity (TPA) as reported by the subjects using the international physical activity questionnaire (IPAQ) (Table 1).

**Table 1:** **Subjects characteristics**.

	**All (n:87)**		**Men (n:46)**		**Women (n:41)**	
	**Mean**	**SD**	**Mean**	**SD**	**Mean**	**SD**
Age	48.75	19.49	48.17	19.39	49.39	19.82
Height^a^	171.90	9.78	179.33	5.94	163.56	5.57
Weight^b^	73.55	13.34	82.47	10.70	63.54	7.71
BMI	24.75	2.98	25.61	2.83	23.78	2.87
Back extension strength^c^	237.26	79.29	288.72	70.18	179.54	38.91
Perceived exertion	4.81	2.53	5.75	2.55	3.75	2.08
IPAQ^d^ TPA^e^	6,201^*^	2,806;	6,119^*^	1,980;	6,624^*^	3,415;
		12,960^*^		12,580^*^		13,070^*^

a *In cm*.

b *In kg*.

c *In Nm*.

d *IPAQ, International physical activity questionnaire*.

e *TPA, Total Physical Activity (in MET/week)*.

* *Median and the 1st and 3rd Quartiles are shown instead of mean and SD*.

The analysis of the trunk angular velocity and acceleration measured using the accelerometer positioned on the lever arm of the Total Trunk device showed minor differences between the concentric and eccentric portions of the exercise. The peak trunk angular velocity was found to be 55.0 ± 0.6°/s for the concentric portion of the exercise and 56.7 ± 0.6°/s for the eccentric portion of the exercise. The peak trunk acceleration was found to be 0.46 ± 0.01°/s^2^ for the concentric portion of the exercise and 0.37 ± 0.01°/s^2^ for the eccentric portion of the exercise.

### RMS-SEMG values for the concentric vs. the eccentric portions of the cyclic exercise

**Figure 4:**
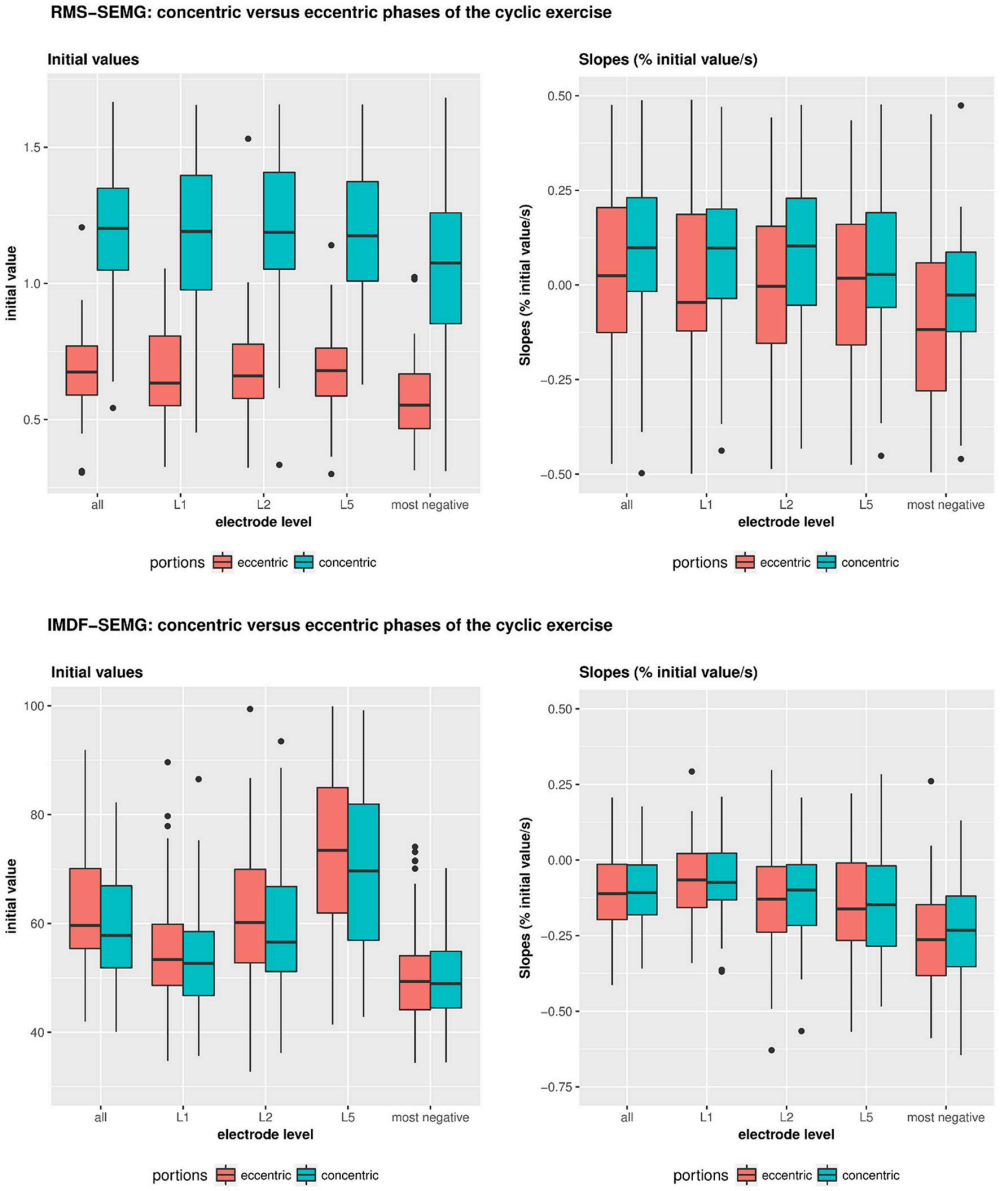
**Illustrates the RMS- and IMDF-SEMG initial values and RMS-&IMDF-SEMG slopes for the concentric and the eccentric portions of the cyclical exercise test**. Initial values and slopes were calculated using regression analyses. The whisker box plots show the median values and the 25 and 75% quantiles and the maximum values.

The RMS-SEMG initial values for the concentric portions of the cyclic exercise were found to be different from the initial RMS-SEMG values for the eccentric portions of the exercise. Table 2 and Figure 4 show the median RMS-SEMG initial values for all muscles for the cyclic exercise test normalized by the RMS-SEMG values derived from the 80% isometric MVC test. The median RMS-SEMG initial values for the concentric portions of the cyclic exercise test were found to be 21–24% higher than the RMS-SEMG values for the 80% isometric MVC test. The same values for the eccentric portions of the cyclic exercise were 32–37% smaller than the RMS-SEMG values for the 80% isometric MVC test. The ratio between the median RMS-SEMG initial values for the concentric vs. the eccentric portions of the exercise was found to range between 1.78 (for the multifidus muscle) and 1.94 (for the iliocostalis lumborum muscle).

**Table 2:** **RMS-SEMG initial values and RMS-SEMG slopes for the concentric and the eccentric portions of the cyclic exercise test**.

	**Concentric**		**Eccentric**		**Concentric vs. Eccentric**	
	**Median**	**Quantile (25%; 75%)**	**Median**	**Quantile (25%; 75%)**	**Test statistic**	***p*-value**
**RMS-SEMG INITIAL VALUES**						
All electrodes	1.21	1.07;1.41	0.67	0.58;0.77	246.25	<0.001^*^
L5 (multifidus)	1.21	1.04;1.44	0.68	0.58;0.76	90.80	<0.001^*^
L2 (longissimus)	1.24	1.07;1.52	0.66	0.57;0.77	96.52	<0.001^*^
L1 (iliocostalis lumborum)	1.22	1.00;1.49	0.63	0.54;0.80	130.83	<0.001^*^
Most negative electrode	1.08	0.85;1.26	0.54	0.44;0.65	91.01	<0.001^*^
**RMS-SEMG SLOPES (% INITIAL VALUE/S)**						
All electrodes	0.132	−0.01;0.34^*^	0.038	−0.14;0.33	3.66	0.048^*^
L5 (multifidus)	0.071	−0.03;0.32^*^	0.055	−0.16;0.30	0.07	0.793
L2 (longissimus)	0.118	−0.05;0.31^*^	−0.002	−0.17;0.25	3.51	0.051
L1 (iliocostalis lumborum)	0.126	−0.02;0.35^*^	−0.002	−0.12;0.30	5.04	0.022^*^
Most negative electrode	−0.027	−0.14;0.09	−0.169	−0.38;0.02^*^	8.72	0.001^*^

*Initial values and slopes were calculated using regression analyses. The table shows the median values and the 25 and 75% quantiles. The table also shows the results of the statistical comparisons between contraction types that were carried out using robust tests based on median comparisons. P < 0.05 are indicated by an asterisk*.

* *p < 0.05*.

During the cyclic exercise, the RMS-SEMG values generally showed significant increases for the concentric portions of the exercise but no such changes for the eccentric portions of the exercise. Table 2 and Figure 4 show the slopes derived via regression analysis of the RMS-SEMG time series for the concentric and the eccentric portions of the cyclic exercise. The table also shows the results of the statistical comparisons of the slopes for the two portions of the cyclic exercise. This analysis revealed significantly more pronounced fatigue-related changes for the concentric portions of the cyclic exercise compared to the eccentric portions of the exercise. This was shown for analyses considering all the electrode sites, only the L5 electrode site, or the electrode site demonstrating the most negative slope.

### IMDF-SEMG values for the concentric vs. the eccentric portions of the cyclic exercise

Table 3 and Figure 4 show the IMDF-SEMG initial values and the slopes derived via regression analysis of the IMDF-SEMG time series. The IMDF-SEMG initial values for both the concentric and eccentric portions of the test revealed the highest value for the iliocostalis lumborum muscle and the lowest value for the multifidus muscle. The IMDF-SEMG initial values for the eccentric portions of the cyclic exercise were generally higher than the same values for the concentric portions of the exercise. However, these differences were not statistically significant. The IMDF-SEMG slopes showed a progressive compression of the frequency content of the SEMG time series toward the lower frequencies. These changes appeared to be more prominent for the longissimus dorsi muscle and the iliocostalis lumborum muscle compared to the multifidus muscle. In addition, these changes appeared to be more pronounced during the eccentric portions of the cyclic exercise than the concentric portions of the exercise. However, these differences were not statistically significant.

**Table 3:** **IMDF-SEMG initial values and IMDF-SEMG slopes for the concentric and the eccentric portions of the cyclic exercise test**.

	**Concentric**		**Eccentric**		**Concentric vs. Eccentric**	
	**Median**	**Quantile (25%; 75%)**	**Median**	**Quantile (25%; 75%)**	**Test statistic**	***p*-value**
**IMDF-SEMG INITIAL VALUES (Hz)**						
All electrodes	57.79	51.85;66.91	59.63	55.39;70.09	0.96	0.325
L5 (multifidus)	69.65	56.91;81.94	73.93	61.91;85.46	1.41	0.247
L2 (longissimus)	56.54	51.15;66.80	60.16	52.75;69.94	2.89	0.073
L1 (iliocostalis lumborum)	52.66	46.75;58.50	53.37	48.60;59.87	0.17	0.687
Most negative electrode	48.93	44.49;54.86	49.31	44.15;54.07	0.05	0.817
**IMDF-SEMG SLOPES (% INITIAL VALUE/s)**						
All electrodes	−0.108	−0.18;−0.02^*^	−0.110	−0.20;−0.01^*^	0.01	0.917
L5 (multifidus)	−0.147	−0.28;−0.02^*^	−0.162	−0.27;−0.01^*^	0.10	0.751
L2 (longissimus)	−0.099	−0.22;−0.02^*^	−0.129	−0.24;−0.02^*^	0.58	0.407
L1 (iliocostalis lumborum)	−0.074	−0.13;0.02^*^	−0.066	−0.16;0.02^*^	0.10	0.759
Most negative electrode	−0.232	−0.35;−0.12^*^	−0.263	−0.38;−0.15^*^	0.73	0.375

*Initial values and slopes were calculated using regression analyses. The table shows the median values and the 25 and 75% quantiles. The table also shows the results of the statistical comparisons between contraction types that were carried out using robust tests based on median comparisons. P < 0.05 are indicated by an asterisk*.

### Effect of age and gender on the RMS-SEMG and IMDF-SEMG ratios for the concentric vs. the eccentric portions of the cyclic exercise

**Figure 5:**
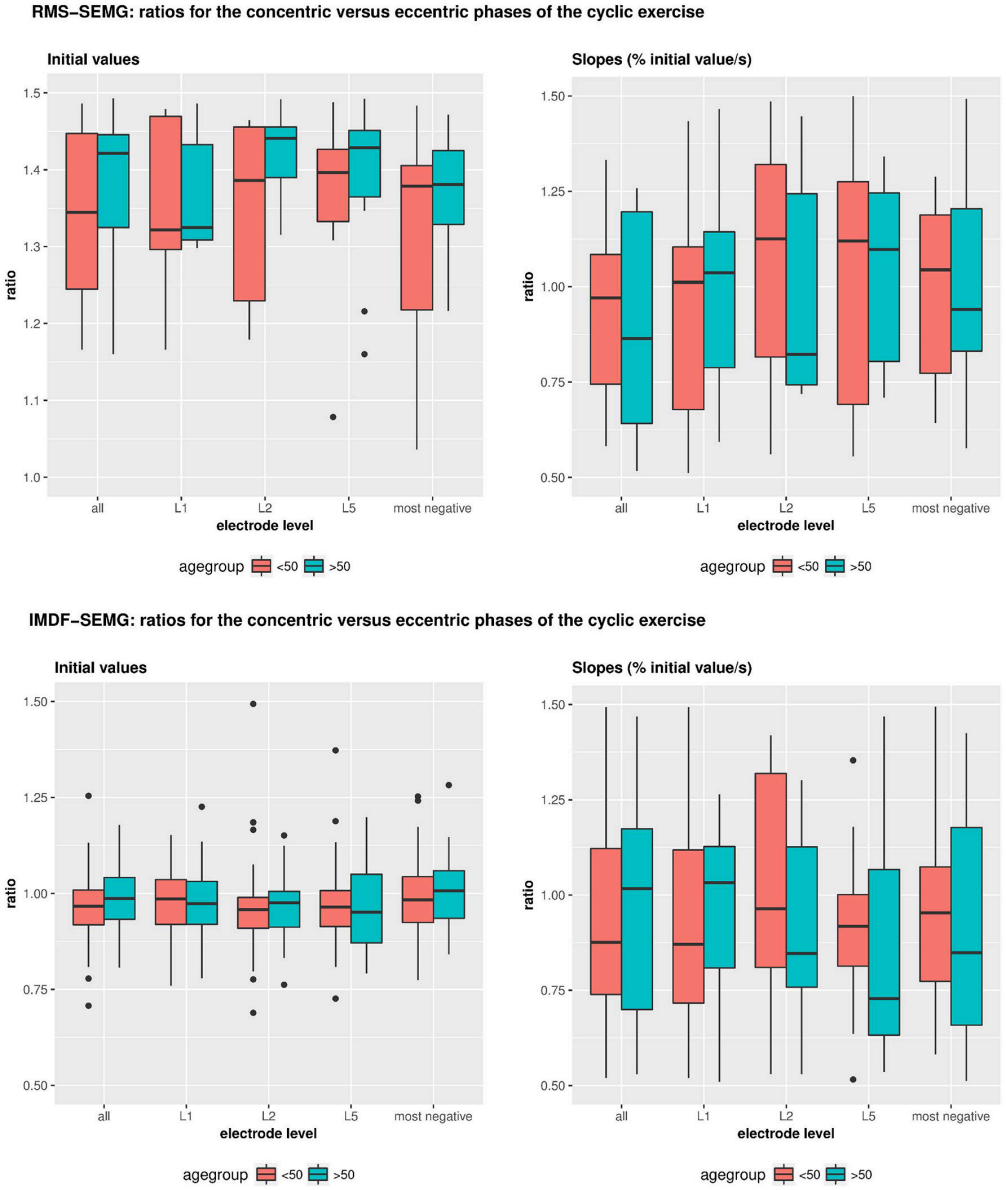
**Illustrates the effects of age and gender on the IMDF-SEMG ratios and RMS-SEMG ratios for the concentric vs. the eccentric portions of the cyclical exercise test**. Data is shown for the initial IMDF-SEMG and RMS-SEMG values as well as the IMDF-SEMG and RMS-SEMG slopes.

Table 4 and Figure 5 show the results of the analyses that we performed to test for potential effects of age and sex on the RMS-SEMG and the IMDF-SEMG ratio values for the concentric vs. the eccentric portions of the cyclic exercise test. We explored potential differences via analysis of the ratios between the RMS-SEMG and IMDF-SEMG initial values for the concentric vs. the eccentric portions of the cyclic exercise. We also analyzed the ratios between the RMS-SEMG and the IMDF-SEMG slopes for the concentric vs. the eccentric portions of the cyclic exercise. We found no statistically significant effect.

**Table 4:** **Effects of age and gender on the IMDF-SEMG ratios and RMS-SEMG ratios for the concentric vs. the eccentric portions of the cyclic exercise test**.

	**IMDF-SEMG**				**RMS-SEMG**			
	**Median (Quantile 25; 75)**	**Age test statistic; *p*-value**	**Gender test statistic; *p*-value**	**Age × Gender test statistic; *p*-value**	**Median (Quantile 25; 75)**	**Age test statistic; *p*-value**	**Gender test statistic; *p*-value**	**Age × Gender test statistic; *p*-value**
**INITIAL VALUES**								
All electrodes	0.97 (0.92; 1.02)	0.39; 0.532	0.44; 0.508	0.50; 0.479	1.85 (1.60; 2.13)	1.33; 0.249	0.41; 0.520	1.04; 0.308
L5	0.96 (0.90; 1.04)	0.81; 0.369	0.17; 0.677	0.35; 0.557	1.81 (1.49; 2.09)	0.15; 0.703	0.92; 0.337	2.73; 0.098
L2	0.96 (0.91; 1.00)	0.46; 0.495	1.14; 0.285	0.11; 0.745	1.89 (1.64; 2.21)	0.62; 0.432	0.44; 0.506	4.15; 0.042
L1	0.98 (0.92; 1.03)	2.01; 0.156	0.71; 0.398	0.37; 0.543	1.83 (1.59; 2.08)	1.28; 0.258	0.02; 0.877	2.82; 0.093
Most negative electrode	0.99 (0.92; 1.05)	5.63; 0.018	1.48; 0.224	0.51; 0.476	1.84 (1.60; 2.27)	0.23; 0.629	0.01; 0.909	3.95; 0.047
**SLOPES**								
All electrodes	0.72 (0.08; 1.16)	1.67; 0.196	1.92; 0.166	0.39; 0.532	0.49 (−0.27; 1.54)	2.45; 0.117	1.07; 0.301	0.05; 0.823
L5	0.68 (−0.06; 1.17)	0.16; 0.690	0.58; 0.444	0.64; 0.423	0.70 (0.03; 1.50)	1.56; 0.212	0.17; 0.683	0.42; 0.519
L2	0.79 (0.34; 1.28)	2.08; 0.150	0.33; 0.568	0.55; 0.457	0.67 (−0.26; 1.51)	0.08; 0.777	0.05; 0.818	0.32; 0.573
L1	0.50 (−0.23; 1.12)	0.59; 0.441	0.73; 0.392	1.21; 0.272	0.41 (−0.31; 1.07)	0.18; 0.670	2.66; 0.103	1.49; 0.222
Most negative electrode	0.92 (0.59; 1.23)	3.95; 0.047	0.40; 0.529	0.25; 0.615	0.37 (−0.16; 0.95)	0.01; 0.919	1.22; 0.270	1.08; 0.298

*Data is shown for the initial IMDF-SEMG and RMS-SEMG values as well as the IMDF-SEMG and RMS-SEMG slopes*.

## Discussion

In this study, we relied upon amplitude and time-frequency parameters derived from the SEMG data to explore differences in the neural control of the back extensor muscles during the concentric vs. the eccentric portions of a cyclic exercise consisting of repetitive trunk extensions. Our findings revealed that: (1) the RMS-SEMG initial values for the eccentric portions of the exercise were considerably lower than the values for the concentric portions of the exercise; (2) the RMS-SEMG slopes generally showed a more pronounced increase in SEMG data amplitude during the concentric vs. the eccentric portions of the exercise; (3) the IMDF-SEMG initial values for the eccentric portions of the exercise were similar to the values observed for the concentric portions of the exercise; (4) the IMDF-SEMG slopes showed a similar compression toward the lower frequencies of the SEMG data for the concentric and the eccentric portions of the exercise; and sex and age did not affect the observed relationship.

It is worth noticing that the RMS-SEMG initial values for the concentric portions of the cyclic exercise exceeded the RMS-SEMG values observed for the 80% MVC tests. It must be noted that the torque generated by the dynamometer during the cyclic exercise was equal to 50% of the force generated by the subjects during the MVC tests. In contrast, the RMS-SEMG initial values for the eccentric portions of the cyclic exercise were approximately 50% of those for the concentric portions of the exercise. Given the quasi-linear relationship between the RMS-SEMG value and the extension force generated by back muscles during isometric contractions (Lariviere et al., 2002; Miura and Sakuraba, 2014), one can observe that the RMS-SEMG initial value for the eccentric portions of the cyclic exercise equated the RMS-SEMG value for an isometric back extension performed at approximately 40–50% MVC. Noteworthy is the fact that, despite the differences in trunk velocity and acceleration between the concentric and eccentric portions of the exercise were minor, slightly larger values of trunk velocity and acceleration were observed during the eccentric portion of the exercise. If the trunk velocity and acceleration during the eccentric portion of the exercise had been equal to the trunk velocity and acceleration during the concentric portion of the exercise, we would have likely observed even lower RMS-EMG values during the eccentric portion of the exercise than the ones observed in the study herein described. These observations appear to be consistent with previous studies showing smaller RMS-SEMG values for eccentric contractions compared to concentric contractions (Bigland-Ritchie and Woods, 1976; Hermann and Barnes, 2001), as well as with studies showing that lower MU firing rates mark eccentric contractions compared to concentric contractions (Duchateau and Enoka, 2008; Kallio et al., 2013). This suggests that differences in excitatory drive as well as in the excitability of the motor neuron pool to the back extensor muscles mark eccentric vs. concentric contractions. Such theory is further corroborated by our findings showing more pronounced fatigue-related RMS-SEMG changes during the concentric compared to the eccentric portions of the cyclic exercise.

The observed differences in RMS-SEMG initial values and respective RMS-EMG slopes for the concentric portions vs. the eccentric portions of the cyclic exercise could be due to differences in firing rate of the same pool of MUs or to differences in the pool of active MUs. In addition, the proportionally lower neuromuscular drive to the motor neuron pool of the back extensor muscles would likely explain why fatigue-related changes in RMS-SEMG were more pronounced during the concentric than the eccentric portions of the exercise. In fact, fatigue-related changes in RMS-SEMG did not occur during the eccentric portions of the exercise. This is consistent with the observation of higher intrinsic force capacity of the muscle fibers during eccentric vs. concentric contractions (Herzog et al., 2015) due an increased contribution of the serial (and parallel elastic) muscle properties in force generation and/or the smaller net force that muscles have to generate when resisting a load that produces an increase in length of the muscles (i.e., eccentric contraction) vs. when moving an equivalent load while muscles are shortening (i.e., concentric contraction). However, analysis of the RMS-SEMG values alone does not allow one to sort out the contributions of these two phenomena to the differences in RMS-SEMG initial values or respective fatigue-related changes observed during eccentric vs. concentric contractions.

The IMDF-SEMG initial values were slightly higher—though the difference was not statically significant—for the lower-effort, eccentric portions of the cyclic exercise compared with the higher-effort, concentric portions of the exercise. This observation is consistent with previous studies with focus on the force vs. MDF-SEMG relationship during isometric back extensions. The MDF-SEMG value was shown to increase as subjects generated larger force outputs during isometric contractions. This increase was observed up to 30% MVC in medial and up to 50% MVC in lateral back extensor muscles. When exceeding these force levels, the MDF-SEMG value was either maintained or showed a slight decrease (Roy et al., 1989; Lariviere et al., 2001; Miura and Sakuraba, 2014). Based on the relationship between the median frequency of the SEMG signal and the muscle fiber conduction velocity, the shape of the action potential and the temperature of the muscle, such atypical changes of the MDF-SEMG with increasing force would be best explained by the recruitment of the in cross section larger slow twitch fibers at lower force thresholds than the smaller fast fatiguing muscle fibers (De Luca et al., 1986; Merletti and Roy, 1996; Knaflitz and Bonato, 1999). Our observation of slightly higher IMDF-SEMG values during the lower-effort, eccentric portions of the cyclic exercise compared to the higher-effort, concentric portions of the exercise further suggests that the relationship between frequency content of the SEMG data and the force output generated by back muscles would not only apply to isometric trunk extensions but would also apply to cyclic exercises of repetitive trunk extensions.

It is important to notice that the non-linear relationship between the IMDF-SEMG value and the force output generated by back muscles during the cyclic exercise of repetitive trunk extensions could also be explained by the simultaneous occurrence of phenomena related to the MU activation physiology of back muscles and factors that may affect the SEMG signal as discussed in the following:

1. Differences in recruitment thresholds and average firing rates mark the MUs of superficial vs. deep back muscles that are likely to be due to their different muscle spindle content (De Luca and Kline, 2012). A close to two-fold difference in number of muscle spindles was observed in the intermediate compared to the medium column of the back extensors (Amonoo-Kuofi, 1982, 1983) as well as in the superficial back extensors of both the medial and the intermediate columns. Deep back muscles appeared to have almost no spindles (Amonoo-Kuofi, 1982). Accordingly, deep back muscles are expected to exhibit higher firing rates and lower MU recruitment thresholds (De Luca and Kline, 2012) than superficial back muscles. Hence, complete MU recruitment for deep back muscles could be achieved already at 50% MVC. This behavior would not be expected in the superficial back muscles which are known to be marked by a high number of muscle spindles.
2. The low-pass filtering effect associated with the soft tissue between the active muscle fibers and the SEMG electrodes affects the characteristics of the SEMG data for deep back muscles more prominently than it affects the characteristics of the SEMG data for superficial muscles (De Luca, 1997). Thus, SEMG data for deep back muscles are marked by lower frequency components than SEMG data from superficial muscles. If all the MUs of deep muscles were recruited at 50% MVC, a further increase in force output generated by these muscles would lead to an increase in the total energy of the SEMG data as more MUAPs would be simultaneously detected using SEMG electrodes. However, the MDF/IMDF value would remain the same. Thus, the spectral representation originating from the deep back muscles would increase the energy in the lower frequency range. This increase might be more prominent than the force-dependent changes in the frequency content of the SEMG data due to the progressive recruitment of high-threshold MUs originating from the superficial muscles. During the eccentric portions of the cyclic exercise, deep back muscles with few muscle spindles would have most or all of their MUs recruited. However, their firing rates would be significantly decreased compared to the rates observed during the concentric portions of the cyclic exercise. This phenomenon would result in a decrease in the energy contributed by deep muscles in the lower frequency range during the performance of the eccentric portions of the cyclic exercise compared to the concentric portions of the exercise.
3. The superposition of MUAPs and the synchronization of the firing rate of concurrently active MUs during the concentric portions of the cyclic exercise might have the effect of decreasing the IMDF-SEMG values. However, this effect would be expected to be small because we collected data using a double-differential technique. Synchronous firing of MUs would be more pronounced in low-force vs. high-force contractions (Kline and De Luca, 2016) or in eccentric vs. concentric contractions (Semmler et al., 2002). Moreover, the influence of MU synchronization may be negligible because synchronous firing seems to occur in less than 10% of muscles (De Luca et al., 1993) and these firing-related effects would affect predominantly the 15–25 Hz range (De Luca, 1997).

Consistently with previous work examining repetitive lifting tasks and trunk extension exercises using training machines (Ebenbichler et al., 2002; Larivière et al., 2010, 2011), we found a clear decrease in the IMDF-SEMG during the concentric portions of the exercise, which is a measure of localized muscle fatigue. A gradual decrease in muscle fiber conduction velocity of the high-threshold MUs is thought to increase the time support of the MUAPs and to “flatten” the waveforms as H^+^ ions accumulate, thereby leading to a compression toward the lower frequencies of the frequency content of the SEMG data (Brody et al., 1991). Unlike the absolute value of the spectral variables, the compression toward the lower frequencies that takes place with fatigue would be minimally affected by spatial filtering. Thus, the decrease in muscle fiber conduction velocity of the deep MUs would likely contribute more to the observed spectral fatigue changes because a proportionally larger number of fast fatiguing motor units would be expected to be active. Moreover, based on the higher than 80% of maximum RMS-EMG during the concentric phase of the contraction, blood supply was likely occluded during the concentric portions of the exercise but partially or even not occluded during the eccentric portions of the exercise. This would suggest that the accumulation or wash out of metabolic correlates of fatigue did not affect the IMDF-SEMG fatigue changes in the concentric portions of the exercise but it did during the eccentric portions of the exercise.

In contrast with previous observations showing a smaller MDF-SEMG slope in low-load compared with high-load sustained isometric back extensions (Roy et al., 1989; Oddsson and De Luca, 2003) and a smaller IMDF-SEMG slope in purely eccentrically-generated vs. purely concentrically-generated maximum knee extensions (Molinari et al., 2006), the findings of this study showed that the IMDF-EMG slopes were similar for the eccentric and the concentric portions of the cyclic exercise. This observation suggests that the high-threshold MUs that are active during the concentric portions of the exercise remain active, at least in significant numbers, during the eccentric portions of the exercise. In the superficial back muscles, however, the high-threshold MUs would likely be de-recruited to a great extent during the eccentric portions of the exercise. Such discrepancy would suggest that the fatigue-related changes in the frequency content of the SEMG data likely reflected fatigue originating from high-threshold MUs of the deep back extensors. These spectral changes of the deep muscles would be low-pass filtered, thus explaining why the decrease in IMDF-SEMG was equally pronounced during the eccentric and the concentric portions of the exercise.

### Implications

The observed patterns of RMS-SEMG and IMDF-SEMG for the eccentric vs. the concentric portions of the cyclic exercise of repetitive trunk extensions examined in the study provide a window of observation on how the CNS controls the MUs of different back muscles during concentric vs. eccentric contractions.

The orderly recruitment of MUs and the common drive of simultaneously active MUs are phenomena that play a role of paramount importance in our interpretation of the results of the study. These phenomena appear to represent the most economic and efficient way the CNS may manage the simultaneous activation, coordination and modulation of MUs activated in the back muscles according to their anatomical location, the length of each muscle and the instantaneous direction and rate of length change when one performs a cyclic exercise consisting of repetitive trunk extensions (Ebenbichler et al., 2002).

Following a descending motor command and the related excitatory input to the motor neuron pool, one would observe a progressive recruitment of back muscle MUs and increase in their firing rate. This is intended to achieve a target force level and movement velocity needed to resist to or move a given external load. In this context, an excitatory feedback from the muscle spindles would facilitate achieving the excitatory input to the motor neuron pool needed to efficiently reach the target force level and movement velocity via mono-synaptic pathways (i.e., type I afferents to the homonymous motor neuron pool) (De Luca and Kline, 2012). In this context, an increase in muscle spindle afferent feedback appears to be mainly due to the slackening and stretching of the muscle spindles resulting from force ripples of unfused MUs (De Luca and Kline, 2012). Accordingly, the deep back extensors with a small number of muscle spindles would be less modulated via the positive feedback from the type I afferent muscle spindles than the superficial and lateral muscles, which are marked by a larger number of muscle spindles.

When comparing concentric vs. eccentric contractions of back muscles, one would observe that the higher intrinsic MU twitch force properties that mark eccentric contractions would result in a slackening effect on the muscle spindles thereby decreasing/inhibiting the excitatory input to the motor neuron pool via mono-synaptic and poly-synaptic pathways (i.e., type Ia and Ib afferents) (Pierrot-Deseilligny and Burke, 2012). This phenomenon would not be observed during concentric contractions. The relative down-regulation of the excitatory input to the motor neuron pool observed during eccentric contractions would predominantly reduce the firing rate of the active MUs of the paravertebral muscles until the maximum recruitment threshold is reached. In contrast, the superficial and lateral back muscles that are marked by high-threshold MUs would be de-recruited and their firing rate would be reduced.

These observations appear to be in agreement with the hypothesis that mono-articular muscles of the back have a different biomechanical role than the multi-articular muscles. The former would have the primary role of improving stability of the back, whereas the latter would have the primary role of coordinating the activity of all muscles recruited for the purpose of achieving the task at hand.

### Limitations

We inferred MU behaviors from the RMS-SEMG and IMDF-SEMG estimates derived from SEMG recordings. Further investigations should be carried out by relying upon techniques suitable to derive the firing rate of active MUs using either invasive recordings or electrode arrays. Future research will have to further explore the MU control properties of superficial and deep back extensors in MU studies. A commercially-available technique was recently utilized in the study of the MU behavior (i.e., firing rate) during cyclic dynamic contractions of muscles of the upper or lower limbs (De Luca et al., 2015). However, it is unclear if this technique would be suitable to reliably study the MUAP trains and fatigue-related changes during cyclic exercises of the back extensors.

One might raise concerns that the level of performance of the cyclic exercise investigated in the study might have biased the results herein presented. It is worth noticing that a significant effort was devoted to instruct and supervise subjects during the performance of the cyclic exercise. Analyses of the data collected using the movement sensors attached to the device utilized in the study to perform the cyclic exercise assured us that the range of motion and the movement velocity of the trunk were virtually the same during the eccentric vs. the concentric portions of the cyclic exercise.

In conclusion the comparison of amplitude and time-frequency parameters of the SEMG data revealed differences in neural control strategies between the concentric and the eccentric portions of a cyclic exercise of repetitive trunk extensions. We propose that such differences be primarily explained by relying upon the concepts of orderly recruitment of MUs and common drive. The observed contraction-specific differences in MU behavior are likely mediated by muscle specific differences in muscle spindle density and related differences in excitatory feedback that might be related to the higher intrinsic twitch force properties of muscles fibers during eccentric contractions compared to concentric contractions.

## Ethics statement

Ethics Committee of the City of Vienna—Number: “EK 11-064-VK-NZ" Full address: Ethikkommission der Stadt Wien Magistratsabteilung 15, Gesundheitsdienst der Stadt Wien 3., Thomas-Klestil-Platz 8 Town Town Tel.: 01/4000-87523.

## Author contributions

GE conceived of the study together with TK and its design, he supervised the study, interpreted the results and provided the first draft the manuscript. TK participated in the study design, carried out the screening of patients, and contributed to drafting the manuscript. LU and SR collected and processed the data and contributed to the discussion of the findings. JK was responsible for technical equipment, preliminary testing and supervision of data quality control and part of the data processing. RH collected data and performed the statistical analyses under the supervision of PM. PB supervised the EMG data processing and PM supervised the statistical analyses. All authors critically revised the manuscript for important intellectual content and gave final approval. PB critically revised the language content of the manuscript.

### Conflict of interest statement

The authors declare that the research was conducted in the absence of any commercial or financial relationships that could be construed as a potential conflict of interest.
